# 2D U‐Net Framework for Multiclass Automated Segmentation of Healthy Structures, Ischemic Stroke, and Glioblastoma in the Rat Brain

**DOI:** 10.1002/nbm.70432

**Published:** 2026-11-04

**Authors:** Sara Ortega‐Espina, Sabela Fernández‐Rodicio, María Luz Alonso‐Alonso, Pelayo García‐Acevedo, Ana Sampedro‐Viana, Marcos Bazarra‐Barreiros, María Teresa Abengoza‐Bello, Antonio J. Mosqueira, Jacobo Porto‐Álvarez, Javier Martínez Fernández, Crhistian‐Mario Oblitas, José Castillo, Pablo Hervella, Ramón Iglesias‐Rey

**Affiliations:** ^1^ Neuroimaging and Biotechnology Laboratory (NOBEL), Clinical Neurosciences Research Laboratory (LINC) Health Research Institute of Santiago de Compostela (IDIS) Santiago de Compostela Spain; ^2^ Department of Functional Biology (Physiology), University of Oviedo Oviedo Spain; ^3^ Neuroradiology Group Health Research Institute of Santiago de Compostela (IDIS) Santiago de Compostela Spain

**Keywords:** 2D U‐Net, AI, artificial intelligence, deep learning, gliobastoma, MRI, segmentation, stroke

## Abstract

Rodents are essential for preclinical brain disease research, including stroke, tumors, and neurodegenerative disorders. Magnetic resonance imaging (MRI) enables longitudinal brain tissue visualization, but accurate segmentation is crucial for meaningful analysis. This study introduces a multitask 2D U‐Net, a convolutional neural network designed for automated segmentation and quantification of brain regions, ischemic stroke lesions, and tumors in rats using T_2_‐weighted imaging (T_2_‐wi) and T_2_‐relaxation maps. The model was evaluated on the following three MRI datasets: (i) segmentation of multiple brain regions in healthy rats, (ii) ischemic stroke lesions using T_2_‐relaxation maps, and (iii) glioblastoma tumors using T_2_‐wi. Performance was assessed using the Dice coefficient (DC), Hausdorff distance (HD), sensitivity (SEN), and volume ratio (VR), comparing network outputs against semiautomatic ground truths and existing models. A total of 1949 images from 111 rats were analyzed, expanded to 5847 through data augmentation. After correcting an animal‐level train/test partitioning that prevented data leakage between subjects, the model achieved robust accuracy across all tasks (DC = 0.80–0.96, HD = 0.83–2.16 mm, SEN = 0.75–0.96, VR = 0.89–1.10), showing a strong correlation (ρ=0.82−0.99) with manual annotations. This framework improves segmentation accuracy, robustness, and applicability across different MRI settings and conditions, addressing issues like interrater variability and time inefficiency when analyzing rodent brain MRI data. Datasets are publicly available at https://github.com/NOBEL‐MRI/Rat‐Datasets.

## Graphical abstract

A multitask 2D U‐Net framework enabling automated, accurate segmentation and quantification of rodent brain regions, stroke lesions, and tumors from MRI data.

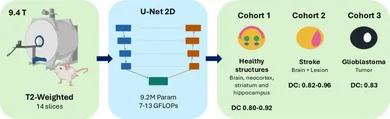

**Abbreviations**
AI: artificial intelligence
CT: computed tomography
DC: Dice coefficient
Dim: dimension
FN: false negative
FP: false positive
HD: Hausdorff distance
MRI: magnetic resonance imaging
ROIs: regions of interest
SEN: sensitivity
T_2_‐wi: T_2_‐weighted imaging
VR: volume ratio

## 1 Introduction

Rodents are widely employed in preclinical research to investigate a range of brain diseases, including stroke, and brain tumors, offering invaluable models for exploring the underlying mechanisms of neurological conditions. In these studies, in vivo imaging technologies, particularly magnetic resonance imaging (MRI), play a vital role in monitoring disease progression, evaluating treatment effects, and gaining insights into the evolving dynamics of neural changes over time [1, 2, 3].

While MRI provides rich and informative brain images, the raw data typically requires segmentation to isolate regions of interest (ROIs) and facilitate quantitative analysis. Segmentation is a crucial step as it allows for the precise identification and measurement of brain structures, lesion volumes, and other key features essential for assessing disease severity and treatment effectiveness, particularly in radiomics analyses [4, 5, 6, 7]. Segmenting these images are especially challenging due to the variability in size, shape, location, and contrast, even within images from the same cohort. This variability often limits the effectiveness of traditional segmentation methods. Furthermore, the small size of rodents makes image acquisition prone to misalignments, which can lead to asymmetric hemispheres in the slices, with a particularly significant impact on anisotropic data. Without proper segmentation, the analysis of MRI data can be inaccurate, prone to interrater and intrarater variability or time‐consuming, highlighting the importance of developing automated, robust methods to streamline this process for enhanced research efficiency [8, 9].

To address these limitations, artificial intelligence (AI), specifically convolutional neural networks like the U‐Net architecture, has emerged as one of the most effective solutions for medical image segmentation [10, 11]. Initially introduced in 2015 for biomedical semantic segmentation, U‐Net has achieved widespread success due to its encoder‐decoder structure with skip connections, which efficiently captures both high‐level contextual information and fine‐grained spatial details [12, 13, 14, 15]. These capabilities have enabled U‐Net to surpass traditional techniques across various imaging modalities (MRI, CT, and microscopy) and applications, including organs, tumors, and lesions [12, 13, 14]. To handle more challenging tasks, numerous adaptations have been developed, such as 3D U‐Net for volumetric data and Attention U‐Net, allowing AI‐driven methods to achieve high accuracy and reliable automation [16, 17, 18, 19, 20, 21, 22]. Building upon these foundations, recent specialized architectures have been proposed for specific pathological applications. For ischemic stroke lesion segmentation, these include FA‐UNet (a FasterNet‐ and attention‐gated hybrid network) [19] and GBA‐NET (a gated bottleneck and attention‐driven architecture for CT and DWI) [21]. Similarly, novel designs have emerged for brain tumor segmentation, such as Mamba U‐Net with region‐wise Bézier intensity augmentation for domain generalization [20] and DiSCNet, a directional split convolution architecture for compute‐efficient diagnosis [22].

In recent years, U‐Net‐based models have gained traction in preclinical rodent research, showing great promise for segmenting complex brain structures [23, 24]. However, current tools remain fragmented. While traditional algorithms (RATS, Sherm) and advanced architectures (Swin‐UNETR) effectively isolate whole brains in healthy animals [25, 26, 27, 28], they lack functionality for internal regions like the striatum or hippocampus [23, 24]. In ischemic stroke models, automated methods focus separately on the entire brain [25], hemispheres [26], or lesions [27, 28, 29], but fail to quantify both the lesion and whole brain simultaneously within a unified pipeline, limiting prognostic accuracy. Similarly, rodent brain tumor research relies on a single standalone 3D U‐Net model constrained by high‐resolution data dependencies, small datasets, and an inability to map affected surrounding regions [30]. Consequently, the primary research gap is not merely a lack of studies, but the absence of a unified framework validated across multiple cohorts (healthy, ischemic, and tumor) to simultaneously segment anatomical and pathological structures. Specifically: (i) internal structure segmentation is extremely scarce and restricted to mice [23, 24]; (ii) whole‐brain extraction tools offer no internal differentiation [31, 32, 33, 34]; (iii) ischemic lesion methods analyze pathologies in isolation without concurrent whole‐brain quantification [25, 26, 27, 28, 29]; and (iv) tumor segmentation remains limited to a single 3D model without regional context [30].

To overcome these collective limitations, our goal is to develop advanced tools that surpass the performance of existing methods while introducing new resources and contributing valuable datasets to the scientific community. Here, we present and validate three 2D U‐Net‐based pipelines enable automatic segmentation and volume quantification using MRI datasets for: (i) multiple ROIs, including the striatum, neocortex, hippocampus, and whole brain from T_2_‐weighted imaging (T_2_‐wi) in healthy subjects; (ii) ischemic stroke lesion volumes and the lesion‐bearing brain using T_2_‐relaxation maps from a transient ischemic stroke model; and (iii) brain tumor lesions using T_2_‐wi from a glioblastoma rat model. We employed four metrics to evaluate the segmentation performance of the proposed frameworks. Additionally, we compared our tools with existing methods to validate their state‐of‐the‐art performance and assess their suitability for preclinical and translational research. By enhancing the accuracy, efficiency, and applicability of brain segmentation or lesion analysis, we aim to provide more reliable and comprehensive resources for preclinical research, ultimately improving the study of neurological diseases and their treatments.

## 2 Materials and Methods

### 2.1 Experimental Animals

All experimental animal procedures were conducted under procedure numbers: 15011/2021/002, 15011/2021/003, 15011/2023/002, and 15011/2021/001 approved by the Animal Care Committee, according to European Union Rules and the Spanish regulation (2010/63/EU and RD53/2013). Animals were kept in a controlled environment at 22°C ± 1°C and 60% ± 5% humidity, with 12:12 h light:darkness cycles, and were fed ad libitum with standard diet pellets and tap water. All surgical procedures and MRI sessions were performed under sevoflurane anesthesia (3%–4%; Abbott Laboratories, IL, USA) delivered in a 70:30 N_2_O:O_2_ mixture as a carrier gas.

### 2.2 Dataset Descriptions

Three distinct in‐house datasets were acquired from three experimental groups of male Sprague–Dawley rats, each weighing between 250 and 350 g. The animals were categorized into Cohort 1 (healthy controls), Cohort 2 (ischemic stroke model), and Cohort 3 (glioblastoma model) based on the data source and specific MRI acquisition protocols.

For Cohort 1 (*n* = 28), healthy rats without any surgical procedures or treatments were selected from the control groups of previous studies. For Cohort 2 (*n* = 33), transient focal cerebral ischemia was induced by transient middle cerebral artery occlusion (MCAo) following previously described surgical procedures [35]. Briefly, using 6‐0 silk sutures, the right external carotid artery as well as the pterygopalatine artery of the internal carotid was ligated. A silicon rubber‐coated size 4‐0 monofilament (diameter 0.19 mm, length 23 mm; diameter with coating 0.37 ± 0.02 mm; coating length 3–4 mm) (Doccol Corporation, Sharon, MA, USA) was inserted into the stump of the right common carotid artery and advanced into the internal carotid artery to 20 mm from the bifurcation to occlude the origin of the MCA. A laser Doppler flow probe (tip diameter 1 mm) attached to a PeriFlux 5000 Laser Doppler Flowmeter (Perimed AB, Stockholm, Sweden) was placed over the thinned skull in the MCA territory (4 mm lateral to bregma) to obtain a continuous measure of relative cerebral blood flow during the experiment. The intraluminal suture was removed 75 min after occlusion to allow reperfusion. Diffusion‐weighted imaging (DWI), magnetic resonance angiography, and T_2_‐wi were acquired 24 h, 7, and 14 days after the experimental intervention.

Finally, for Cohort 3 (*n* = 50), glioblastoma multiforme was induced by stereotaxic implantation of the F98 cell line [36]. After anesthesia, animals were then mounted onto a stereotaxic frame. A midline incision was performed, and a burr hole was punctured using a 16‐gauge needle. The burr hole coordinates were, using the bregma as a reference point, 1 mm anterior and 2.5 mm lateral to the right. Then 1 × 10^5^ cells suspended in a volume of 10 μL of nonsupplemented cell culture medium (Dulbecco's Modified Eagle Medium [DMEM], Gibco, Invitrogen; Paisley, UK) were injected into the brain with a 26‐gauge needle at a depth of 4.5 mm from the surface of the skull (injection rate of 2 μL/min). MRI measurements were evaluated at 7, 14, and 21 days after the surgery.

### 2.3 MRI Experiments

MRI studies were conducted on a 9.4 T horizontal bore magnet (Bruker BioSpin, Ettlingen, Germany) with 12 cm wide actively shielded gradient coils (440 mT/m). Radiofrequency transmission was achieved using a birdcage volume resonator, and the signal was detected using a four‐element arrayed surface coil positioned over the head of the animal. The latter was fixed using a tooth bar, earplugs, and adhesive tape. The transmission and reception coils were actively decoupled from each other. Gradient echo pilot scans were performed at the beginning of each imaging session to position the animals accurately inside the magnetic bore.

For Cohorts 1 and 2, T_2_‐wi were acquired using the multislice multispin‐echo (MSME) sequence to examine brain anatomical regions or determine infarct volume at 24 h, 7, and 14 days after the occlusion. Meanwhile, T_2_‐wi were obtained using the rapid acquisition with relaxation enhancement (RARE) sequence for tumor quantification 7, 14, and 21 days after the surgery. A complete overview of MR scans, along with a summary of the main imaging acquisition parameters, has been compiled (Table 1).

**TABLE 1:** Summary of MRI scanning parameters.

MRI acquisition	T_2_‐wi MSME	T_2_‐wi RARE
Parameters	Sequence	Sequence
Repetition time (ms)	3000	2500
Echo time (ms)	9	33
Number of echoes	16	1
Flip angle (°)	180	90
Number of averages	2	3
Field of view (mm^2^)	19.2 × 19.2	25.6 × 25.6
Matrix	192 × 192	256 × 256
Number of slices	14	14
Slice thickness (mm)	1	1
In‐plane pixel size (mm)	0.1	0.1
Interslice gap (mm)	No	No
Bandwidth (KHz)	75	37
Orientation	Axial	Axial
Acquisition time (minutes)	19	4

### 2.4 Manual MRI Annotation and Ground Truth Generation

Ground truth masks for all three cohorts were generated via slice‐by‐slice manual segmentation performed by two trained observers (SOE and SFR) using ImageJ software (National Institutes of Health, Bethesda, MD, USA). To evaluate the raw generalization capability of the proposed U‐Net, no prior MRI preprocessing steps, such as bias‐field inhomogeneity correction, artifact removal, spatial registration, or skull stripping, were applied to the structural T_2_‐wi datasets (Figure 1). For Cohort 1, the whole brain, hippocampus, striatum, and neocortex were manually identified, delineated, and quantified on T_2_‐wi, averaged across the 4th to 8th echoes, using a standard murine atlas as a reference (https://scalablebrainatlas.incf.org). For Cohort 2 (ischemic stroke), regions of hyperintense T_2_ signals corresponding to ischemic tissue were manually delineated on quantitative T_2_‐relaxation maps computed from the multiecho T_2_‐wi sequences [35]. In this cohort, the total brain volume was also segmented to isolate it from surrounding nonbrain structures. For Cohort 3, tumor margins were segmented, and neoplastic volumes were quantified directly from the T_2_‐wi RARE images. In all cases, region volume, infarction volume, T_2_‐relaxation time, or tumor volume were calculated by multiplying the selected regions by the voxel size or signal.

**FIGURE 1:**
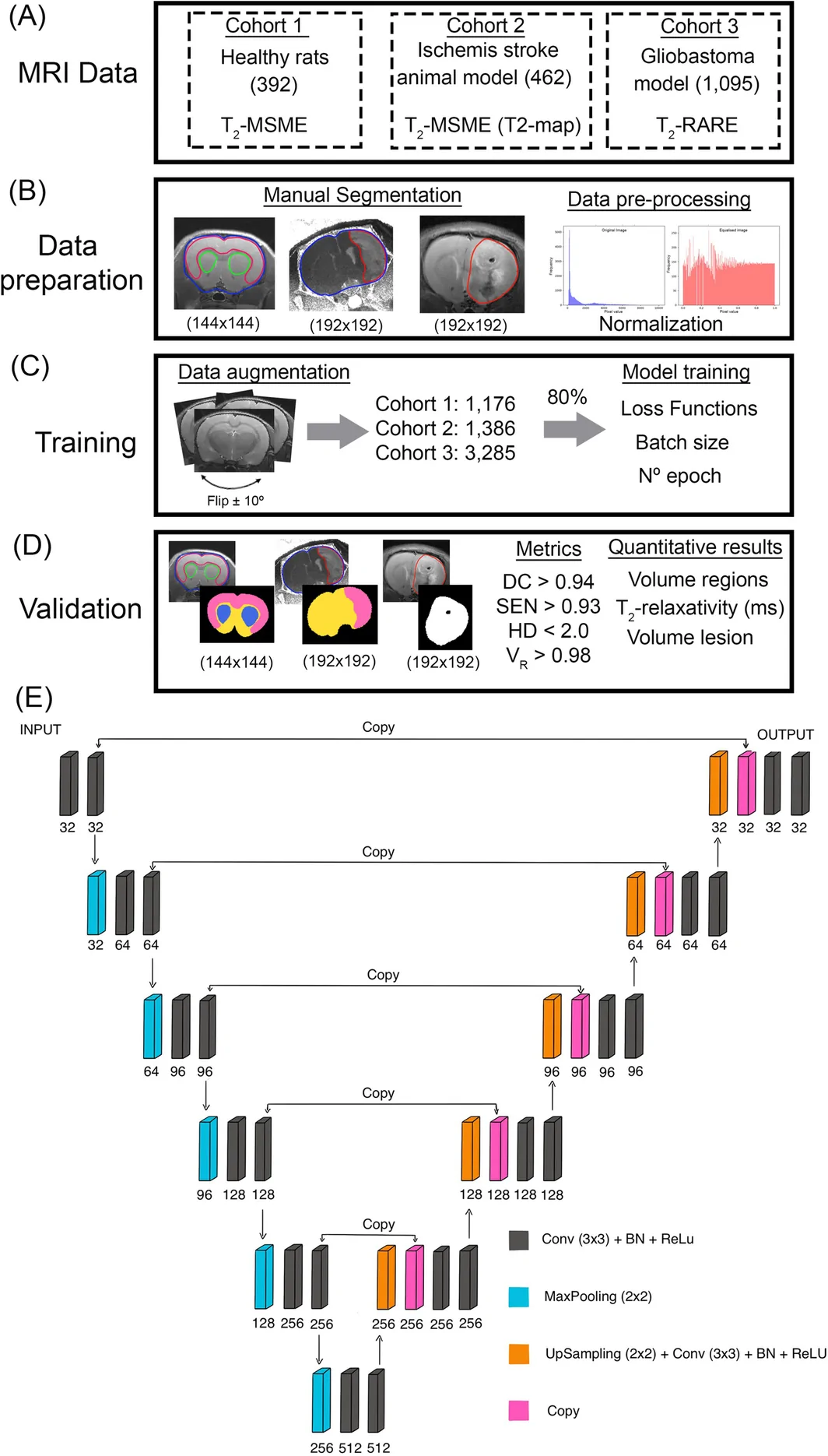
Schematic workflow and the proposed 2D U‐Net architecture. The overall pipeline encompasses: (a) raw MRI data acquisition, (b) manual expert annotation (ground truth) and image preprocessing, (c) data augmentation and model training, and (d) performance validation. (e) The 2D U‐Net architecture comprises a contracting encoder path to capture contextual features and a symmetrical expanding decoder path for precise spatial localization. The network accommodates distinct MRI modalities as inputs (T_2_‐wi or T_2_‐relaxation maps) to generate the corresponding multiclass structural or pathological segmentation masks as outputs.

### 2.5 Automated MRI Analysis Using U‐Net

Data preparation, tensor formatting, and automated evaluation pipelines for all three cohorts (T_2_‐wi for Cohort 1, T_2_‐relaxation maps for Cohort 2, and T_2_‐wi RARE for Cohort 3) were fully automated using custom Python scripts. The implementation was carried out with Python (V 3.8.1), and the following libraries were used: NumPy (V 1.26.4) [37], Tifffile (V 2023.4.12), Nibabel (V 5.3.0), SciPy (V 1.13.1) [38], Skimage (V 0.23.2), Matplotlib (V 3.8.4) [39], Keras (V 3.6.0), and Tensorflow (V 2.17.0). Modular construction has been used to implement all the quantification methods. As a result, it was possible to easily replace existing stages or add new ones to the quantification workflow.

### 2.6 U‐Net Architecture

The selected architecture for this task was U‐Net, a model that has become widely adopted for biomedical segmentation tasks due to its effectiveness and efficiency. Initially introduced by Ronneberger et al. in 2015 [11], U‐Net is particularly well‐suited for image segmentation in medical imaging, where precise delineation of structures is crucial. For this study, a 2D U‐Net model with considerable depth was chosen over a 3D implementation to reduce computational time (Figure 1f). Each step of the encoder path consists of a convolutional layer with a 3×3 kernel, followed by a batch normalization layer and 2×2 max pooling. The decoder path is quite similar, with the addition of 2×2 up sampling layers instead of max pooling layers. Batch normalization layers have been applied to accelerate training and improve model stability by normalizing the input of the preceding layer [40].

In two out of the three cohorts, the segmentation was multiclass, meaning the MRI is processed to generate a colored map where each region corresponds to a distinct class. This process is typically handled by the Softmax (Equation (1)) activation function [41], which assigns each pixel to one of the multiple classes. In contrast, for binary classification tasks, such as glioblastoma identification, the Sigmoid activation function *σ*(*x*) was used (Equation (2)). This results in a prediction that indicates whether a given pixel belongs to the target class (glioblastoma) or not [42].

(1) $$\text{Softmax}\;\left(x_{i}\right)=\frac{e^{x_{i}}}{\sum_{j}e^{x_{j}}}$$

(2) $$\;\sigma \left(x\right)=\frac{1}{1+e^{-x}}$$

where *x*_i_ represents the logit or raw score for the *i*‐th class, which is generated by the model's output layer. In the context of the softmax function, the indices i and j ensure that all classes are taken into account during the computation.

Another activation function used in both the encoder and decoder paths of the model was the Rectified Linear Unit *f*(*x*) (Equation (3)), which primarily serves to convert values below zero into zero [43]:

(3) $$f\left(x\right)=\{\begin{array}{c} x_{i},\;\text{if}\;x_{i}\geq 0\\ 0,\;\text{if}\;x_{i}<0 \end{array}$$

### 2.7 Data Preprocessing and Data Augmentation

To minimize contrast variations arising from differing imaging conditions and enhance model convergence, a two‐step intensity normalization pipeline was applied to all datasets prior to network training. First, local histogram equalization was performed on each slice to optimize localized contrast distributions and reduce regional signal intensities caused by magnetic field inhomogeneities. Second, global intensity values were mapped to a standard bounding interval [0, 1] utilizing a pixel‐wise Min–Max scaling operation.

Data augmentation techniques used included a random rotation of ±10° for each image to mimic the variations in animal positioning during MRI scanning. This strategy tripled the size of the training subset only; augmentation was applied after partitioning each cohort into training and test sets at the animal level, and was never applied to the test set, to avoid information leakage between augmented derivatives and their source images. This approach enhanced the model's robustness by simulating natural variability in animal positioning during MRI, improving generalization to small spatial misalignments with minimal preprocessing complexity. We acknowledge as a limitation that only rotation was systematically evaluated; other augmentation strategies (e.g., translation, shearing, elastic deformations, or gamma/intensity transformations) were not tested and are left as future work under the corrected subject‐level partitioning scheme.

### 2.8 Model Training and Validation: Loss Functions, Batch Size, and Number of Epoch

All training and inference processes were performed on a workstation equipped with an MSI desktop running Windows, featuring a 13th generation Intel(R) Core (TM) i7‐13700F CPU at 2.10 GHz, 16 GB of RAM, and an NVIDIA GeForce RTX 3060 GPU with 8 GB of dedicated memory. This setup provided sufficient computational power to efficiently train the 2D segmentation models and perform inference within practical timeframes.

To train the proposed U‐Net model the training and test sets were partitioned at the animal (subject) level rather than at the image level: for each cohort, 80% of the animals were randomly assigned to the training set and the remaining 20% to an independent test set, using a group‐based partitioning strategy (scikit‐learn's GroupShuffleSplit, random_state = 42) that guarantees no slice from a given animal appears in both subsets. Data augmentation was applied exclusively to the training subset, after this partition, so that no augmented derivative of a test‐set image was ever seen during training. Model checkpoints were selected based on the minimum training loss; no early‐stopping or checkpoint‐selection criterion based on test‐set performance was used. Hyperparameters (loss function, learning rate, batch size, and number of epochs) were fixed per cohort from empirical pilot runs. A summary of the total number of subjects and samples, excluding data augmentation, along with key hyperparameters for training, including the number of epochs, learning rate, and batch size, was provided (Table 2).

**TABLE 2:** Summary of training parameters.

Dataset	*n*	Batch size	No. epoch	Learning rate
Cohort 1	28	8	60	0.001
Cohort 2	33	16	60	0.0005
Cohort 3	50	8	150	0.001

Loss functions play a crucial role in optimizing the model's parameters during training [39], and are essential for its proper development. Various loss functions are tailored to different types of problems, and for image segmentation tasks like ours, functions such as dice loss, weighted dice loss, binary cross entropy (for binary segmentation), categorical cross entropy (for multiclass segmentation), and Tversky focal loss [44] have been tested.

When selecting a loss function, factors such as the nature of the problem and the potential for class imbalance must be carefully considered. In the case of ischemic stroke or glioblastoma lesions, disease progression is often unpredictable, leading to inherent class imbalance.

For Cohort 1, categorical cross entropy was employed, as it is commonly used in multiclass segmentation (Equation (4)). This loss function predicts a probability for each class present in the true label. For Cohort 2, the Tversky Index with α = 0.7 (minimized as Tversky Loss = 1 − Tversky Index) was used (Equation (5)). This function introduces two hyperparameters that control the weight of false positive‐ (FP) and false‐negative (FN) errors, effectively addressing class imbalance [45]. Tversky Loss was chosen over Dice Loss for stroke segmentation due to its ability to better manage class imbalance and small lesion size by weighting false negatives more heavily, thereby improving sensitivity and reducing undersegmentation. In Cohort 3, binary cross entropy was applied (Equation (6)), which measures the difference between the predicted probabilities and the actual binary labels (0 or 1).

(4) $$L\left(p,y\right)=-\sum_{i}y_{i}\cdot \log\left(p_{i}\right)$$

where *p* is the predicted probability that the sample belongs to the positive class, and *y* is the actual label (0 or 1) of the sample.

(5) $$\text{Tversky Loss}=1‐\frac{\mathrm{TP}}{\mathrm{TP}+\mathrm{\alpha FN}+\left(1-\alpha \right)\mathrm{FP}}$$

where TP stands for true positives, and α is a hyperparameter that adjusts the weight of FNs (the higher the α the more the FNs are penalized). Formally, *β* = 1 − *α* weights false negatives; with *α* = 0.7 (*β* = 0.3), false negatives are penalized more heavily than false positives, favoring sensitivity and reducing undersegmentation of the lesion, consistent with the values reported in Results.

(6) $$L\left(p,y\right)=-\left[y\cdot \log\left(p\right)+\left(1-y\right)\cdot \log\left(1-p\right)\right]$$

### 2.9 Performance Evaluation Metrics: Dice Coefficient, Sensitivity, Hausdorff Distance, and Volume Ratio

Four metrics were used to assess the segmentation performance of the proposed frameworks: the: Dice coefficient (DC), Hausdorff distance (HD), sensitivity (SEN), and volume ratio (VR). DC is one of the most widely used metrics in segmentation problems, both binary and multiclass (Equation (7)). Broadly speaking, it measures the similarity between the ground truth and the prediction. The overlapping area between them is proportional to this value, which is always between 0 and 1. Sensistivity measures the ability of a model to correctly identify positive cases within a dataset (Equation (8)). HD measures the maximum of the minimum distances between points of two sets (Equation (9)) [46]. VR provides a measure of the ratio between the volume predicted by the model and the corresponding ground truth volume (Equation (10)). No cross‐class aggregation into a single composite score was performed: DC, SEN, HD, and VR are each reported independently per anatomical structure or pathological target (striatum, neocortex, hippocampus, and brain in Cohort 1; lesion and brain in Cohort 2; tumor in Cohort 3). For DC and SEN, slices in which a given class was absent from the ground truth were excluded from that class's statistics, to avoid degenerate 0/0‐type values; for HD, slices in which the class was present in only one of the ground truth or the prediction were likewise excluded.

(7) $$\text{Dice Coefficient}=\frac{2\left|A\cap B\right|}{\left|A\right|+\left|B\right|}$$

(8) $$\text{Sensitivity}=\frac{\mathrm{TP}}{\left(\mathrm{TP}+\mathrm{FN}\right)}$$

where A corresponds to the ground truth and B the predicted segmentation mask.

(9) $$\text{Hausdorf Distance}=\max\;\left\{\max_{a\in \partial A}\min_{b\in \partial B}|b-a|,\;\max_{b\in \partial B}\min_{a\in \partial A}|a-b|\right\}$$

where the formula calculates the maximum, across both directions, of the minimum Euclidean distance from each point on one boundary to its nearest neighbor on the other—that is, the classical (nonaveraged) HD, not an average of point‐wise distances. Pixel distances were converted to millimeters using the in‐plane pixel spacing derived from the field of view and acquisition matrix (0.1 mm/pixel for both the MSME and RARE sequences; Table 1).

(10) $$\text{Volume ratio}=\frac{V_{\text{Predicted}}}{V_{\text{Ground Truth}}}$$

These metrics collectively enable us to communicate the model's confidence and reliability to end users. For instance, low sensitivity values in certain cases may indicate undersegmentation, particularly for small or poorly defined lesions, such as those observed in early‐stage ischemic stroke. Conversely, discrepancies in VR or DC often point to oversegmentation, especially when nonspecific hyperintensities are present. The HD proves especially valuable for detecting boundary‐level inaccuracies, which tend to occur in lesions with irregular or infiltrative margins, such as glioblastomas. Although these boundary deviations may not substantially impact volume‐based metrics, they are critically important for applications that require accurate and detailed lesion delineation.

## 3 Results

### 3.1 Implementation

The analysis was developed using a Python script, and the datasets are available for download at https://github.com/NOBEL‐MRI/Rat‐Datasets. Across the three experimental cohorts, a total of 111 animals were evaluated, yielding 1949 original 2D MRI slices. Following the threefold data augmentation expansion, the definitive volumetric matrix comprised 5847 images. To prevent data leakage between the training and test sets, splitting was performed at the animal level, that is, all (augmented) slices originating from a given animal were assigned exclusively to either the training or the test subset—rather than at the level of individual slices, as follows: (i) Cohort 1 (anatomical target): comprising 28 healthy subjects (392 baseline slices before augmentation), the dataset was split into 22 animals / 308 slices for network training and six animals /84 slices for independent testing. (ii) Cohort 2 (ischemic stroke): comprising 33 subjects monitored across longitudinal phases, 11 animals at 24 h, 10 animals at 7 d, and 12 animals at 14 d after stroke (462 baseline slices before augmentation), the dataset was split into 26 animals/364 slices for training and seven animals /98 slices for independent testing. (iii) Cohort 3 (glioblastoma): comprising 50 subjects evaluated at progressive oncological stage: 24 animals at 7 days, 17 animals at 14 days, and nine animals at 21 days after induction (1095 raw slices before augmentation). The dataset was likewise split at the animal level, with 33 animals reserved for independent testing, with 163 total across different timepoints. Slice counts reported above are prior to the threefold augmentation, which was applied only to the training subset after splitting.

To maximize semantic diversity and optimize feature extraction across varying pathological stages, slices from all experimental time points within Cohorts 2 and 3 were pooled prior to partitioning. A detailed overview of the core image characteristics, tensor dimensionalities, and storage formats (such as NIfTI and TIFF) for both the input modalities and target ground truth masks across all cohorts is summarized in Table 3. These standardized specifications ensure full experimental reproducibility and facilitate benchmarking across alternative preclinical segmentation frameworks.

**TABLE 3:** Summary of datasets.

	Input			Output		
Dataset	Image	Format	Size	Mask	Format	Size
Cohort 1	T_2_‐wi	Tiff	192 × 192	Striatum, neocortex Hippocampus, brain	Tiff	192 × 192 × 5
Cohort 2	T_2_‐map	Tiff	192 × 192	Lesion, brain	Tiff	192 × 192 × 3
Cohort 3	T_2_‐wi	Tiff	144 × 144	Tumor	Nifti	144 × 144 × 1

### 3.2 Training Process

The training phase was conducted with consideration of batch size, number of epochs, and learning rate, specifically tailored for each cohort. The number of epochs varied across cohorts, ranging from 60 to 150, with a maximum batch size of 16 used (Table 2). Despite these differences, the selected number of epochs proved to be the most effective for training, allowing for adequate model convergence and optimizing performance tailored to each specific dataset. The learning rate exhibited more consistency across cohorts, with values typically set to 0.001 or 0.0005. The selected values were critical for ensuring efficient convergence during training, helping the model avoid issues such as overshooting the optimal solution or excessive training time. By carefully selecting the learning rate for each cohort, a balance was achieved between training speed and the model's ability to generalize effectively to new, unseen data.

Brain region segmentation and ischemic stroke lesions require multiclass segmentation because these studies often aim to measure not only the presence of a lesion but also its extent relative to healthy tissue. Multiclass segmentation allows for quantitative comparisons of different tissue types, making it more suitable than binary segmentation approaches. Addressing class imbalance is crucial for unbiased model performance. Different loss functions were applied for optimal results in each cohort. Categorical cross entropy was used for brain region segmentation in Cohort 1, effectively handling multiclass classification. Tversky loss was chosen for ischemic stroke segmentation in Cohort 2 to balance false positives and false negatives. Cohort 3, focused on glioblastoma, employed binary cross entropy for robust lesion detection. Additionally, batch normalization layers were included in all models to stabilize training, improve convergence speed, and enhance generalization across different segmentation tasks.

### 3.3 Model Optimization and Network Training

The training phase was executed by dynamically tailoring hyperparameter configurations including batch size, epoch limits, and initial learning rates, to the distinct structural complexities of each experimental cohort (Table 2). The optimization window varied between 60 and 150 epochs, utilizing a maximum mini‐batch constraint of 16 slices to balance memory bandwidth and gradient stability. This cohort‐specific grid allowed for adequate stochastic convergence while preventing overfitting. The initial learning rate ($L_{R}$) exhibited high structural consistency across models, optimized at either 0.001 or 0.0005 via the Adam optimizer. These targeted boundary values were critical for ensuring efficient gradient descent trajectories, mitigating the risk of sharp objective function overshooting while minimizing computational training overhead. By systematically balancing the $L_{R}$ and batch size profiles for each distinct cohort, an optimal trade‐off was struck between training acceleration and out‐of‐sample architectural generalizability. Furthermore, to stabilize internal covariate shift, accelerate backpropagation convergence velocities, and enhance multitask spatial generalization, batch normalization layers were strategically integrated after each convolutional block throughout the entire framework.

Quantitative neuroimaging workflows for anatomical mapping (Cohort 1) and ischemic injury (Cohort 2) necessitate robust multiclass segmentation topologies. Unlike restrictive binary systems, multiclass frameworks simultaneously map the spatial extent of a pathological lesion alongside its exact volumetric ratio relative to surrounding healthy parenchyma, enabling multiparametric tissue characterization. To mitigate severe pixel‐wise class imbalances, where healthy background voxels mathematically overwhelm localized target structures—specialized objective loss functions were customized for each experimental paradigm: (i) Anatomical multiclass segmentation (Cohort 1): To drive multiclass pixel classification across the striatum, neocortex, and hippocampus, a categorical cross‐entropy loss function was deployed, optimizing multiprobability distributions across distinct structural targets. (ii) Ischemic stroke delineation (Cohort 2): To isolate amorphously shaped ischemic penumbras and mitigate voxel undersampling, the Tversky Loss function was implemented. This formulation introduces tunable hyperparameter alpha and beta weights to strictly balance false positives and false negatives, prioritizing edge‐tracking recall over symmetric voxel overlap. (iii) Glioblastoma neoplastic identification (Cohort 3): For robust focal lesion detection and solid mass isolation, a stabilized binary cross‐entropy objective function was utilized, maximizing binary boundary margins between the core oncological mass and the remaining healthy tissue.

### 3.4 Computational Efficiency and Qualitative Segmentation Outputs

The automated pipeline demonstrated high computational efficiency during the deployment phase; the trained 2D U‐Net achieved an average inference time of 0.2–0.3 s/sample on the target workstation. This rapid processing speed enables near‐instantaneous volumetric quantification, making it highly suitable for high‐throughput preclinical screening.

Beyond inference speed, model efficiency was further characterized in terms of parameter count, computational cost, and GPU memory footprint for the three trained networks. All three models share nearly identical architectures, differing only in the number of output classes and input resolution: the Cohort 1 (healthy structures) and Cohort 2 (ischemic stroke) networks operate on 192 × 192 inputs (9,162,245 and 9,162,179 parameters, respectively; 13.02 and 13.01 GFLOPs), while the Cohort 3 (glioblastoma) network operates on a smaller 144 × 144 input (9,162,113 parameters; 7.25 GFLOPs). Peak GPU memory usage, measured on an NVIDIA T4 GPU using an isolated, freshly restarted runtime per model with a warm‐up inference pass to avoid transient memory‐allocator effects, was 108.9 MB (Cohort 3), 109.7 MB (Cohort 2), and 109.7 MB (Cohort 1). This consistency across cohorts, despite the different output‐class counts and input resolutions, confirms the lightweight and predictable computational footprint of the proposed 2D architecture, supporting its suitability for deployment on standard laboratory workstations without specialized high‐end GPU hardware.

To assess the contribution of intensity histogram equalization as a preprocessing step, an ablation study was performed by retraining each cohort's model on nonequalized images and comparing its performance on the same animal‐level test partition. For Cohort 2 (ischemic stroke) and Cohort 3 (glioblastoma), removing equalization led to a moderate reduction in segmentation accuracy relative to the equalized models, without qualitatively altering the overall pattern of results. For Cohort 1 (healthy structures), removing equalization produced a markedly different behavior: under standard inference (using the batch normalization statistics accumulated during training), test‐set DC collapsed to near‐zero for the neocortex, striatum, and hippocampus, despite the nonequalized model reaching comparable training‐time metrics (DC: 0.93–0.96) to the equalized model. This behavior was traced to instability in the batch normalization layers' running statistics, driven by greater interimage intensity variability in the absence of equalization. Forcing batch‐wise (rather than running‐average) normalization statistics during test‐time evaluation recovered performance to DC 0.89 (brain), 0.89 (neocortex), 0.672 (striatum), and 0.85 (hippocampus), comparable to, though for most structures slightly below, the equalized model's corresponding values (0.92, 0.93, 0.80, and 0.82). We report this diagnostic evaluation for completeness, while noting that it relies on a nonstandard test‐time normalization protocol rather than the trained model's default inference behavior; taken together, these results support histogram equalization as a stabilizing preprocessing step for Cohort 1, and as a moderately beneficial one for Cohorts 2 and 3.

Segmentation performance was also stratified by structure or lesion‐volume tercile to assess whether accuracy varied systematically with target extent (Supplementary Table 1). For Cohort 1 (healthy structures), animals were divided into size terciles (two animals per tercile) separately for each structure. Unlike the pathological cohorts described below, no consistent, monotonic relationship between structure size and segmentation accuracy was observed for the striatum, neocortex, or hippocampus: DC remained stable across terciles for all four structures (e.g., striatum: 0.803, 0.821, and 0.773 in the small, medium, and large terciles, respectively; hippocampus: 0.831, 0.816, and 0.827). This is consistent with the substantially narrower range of anatomical variation in healthy structures across animals compared with pathological lesions: for example, striatum volume ranged from 83 to 106 mm^3^ and hippocampus volume from 108 to 115 mm^3^ across terciles in this cohort, in contrast to the order‐of‐magnitude variation typically seen in lesion volumes. Given the small number of animals per tercile (*n* = 2), these results should be interpreted as exploratory rather than conclusive. For Cohort 2 (ischemic stroke), the same stratification was performed by lesion‐volume tercile (*n* = 7 test animals in total, so each tercile contains only two to three animals). In small lesions (*n* = 3): DC = 0.79 ± 0.24, SEN = 0.82 ± 0.26, HD = 1.09 ± 0.67 mm, VR = 1.02 ± 0.03. In medium lesions (*n* = 2): DC = 0.83 ± 0.19, SEN = 0.83 ± 0.21, HD = 0.97 ± 0.89 mm, VR = 0.96 ± 0.04. In large lesions (*n* = 2): DC = 0.86 ± 0.20, SEN = 0.85 ± 0.22, HD = 1.00 ± 1.05 mm, VR = 0.97 ± 0.04. Unlike the healthy structures in Cohort 1, performance here was consistently lower in small lesions than in medium or large lesions. For Cohort 3 (glioblastoma), lesion‐volume tercile stratification (*n* = 33 test animals, 11 per tercile) showed VR = 1.28 ± 0.98 in small lesions, 1.04 ± 0.11 in medium lesions, and 0.98 ± 0.11 in large lesions. As in Cohort 2, the model was highly consistent in medium and large lesions, with variability concentrated almost entirely in small lesions. Taken together, the ischemic stroke and glioblastoma cohorts show a consistent pattern of reduced accuracy and greater variability for the smallest lesions, whereas no comparable size‐related effect was observed for the healthy anatomical structures in Cohort 1, likely reflecting their far narrower range of size variation across animals.

Representative qualitative segmentation outputs across the three experimental cohorts are presented in Figure 2, illustrating the raw input MRI, the normalized image tensor, and a direct visual comparison between the manual expert ground truth and the network's predicted masks. For Cohort 1, the network accurately resolved the fine anatomical boundaries of internal structures, including the hippocampus, striatum, and neocortex. For Cohort 2, the framework successfully isolated the ischemic stroke lesion alongside the remaining brain parenchyma, whereas for Cohort 3, the predicted masks tightly delineated the dense tumor mass.

**FIGURE 2:**
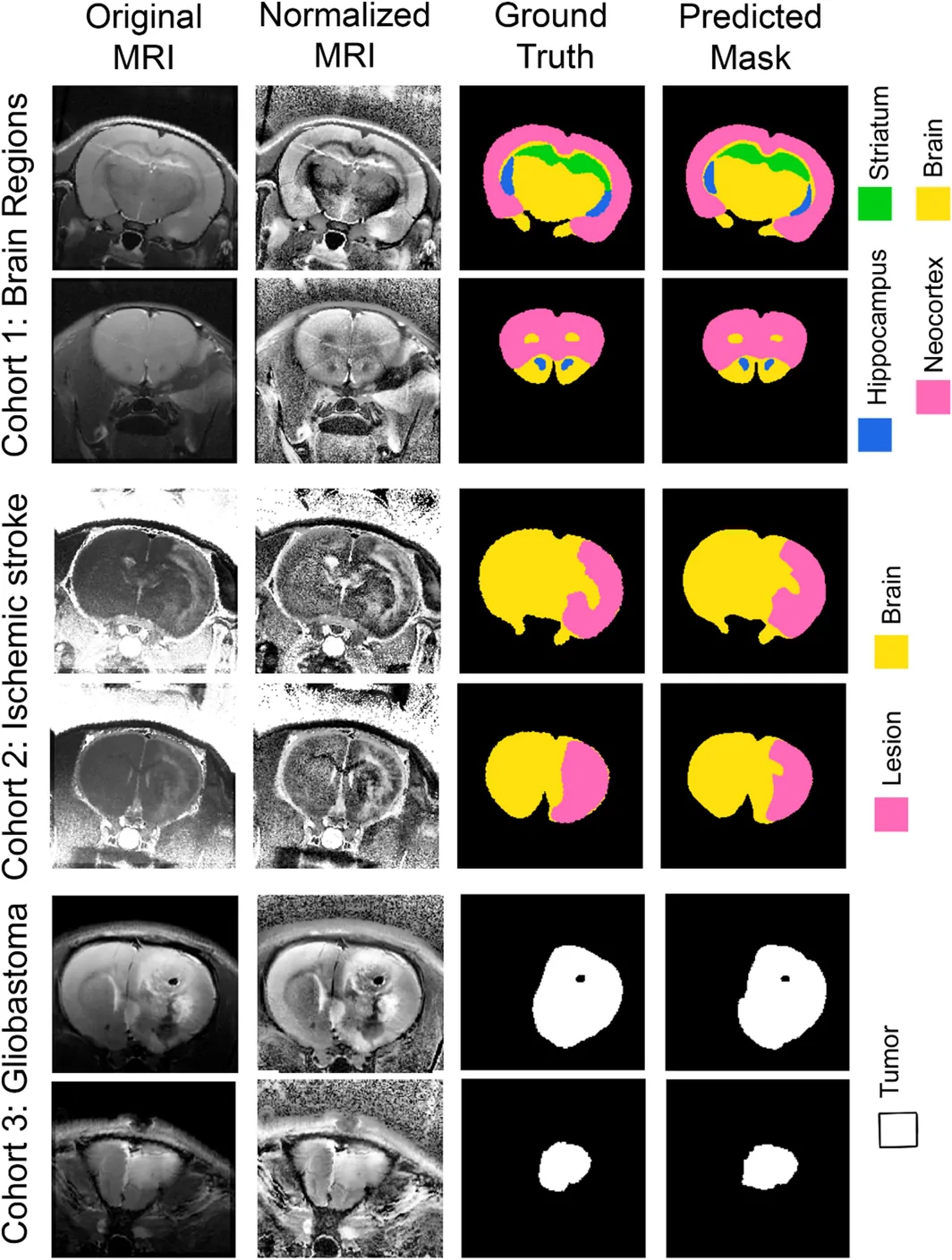
Qualitative segmentation performance across the three experimental cohorts. From left to right, each panel showcases: the raw input MRI slice, the corresponding preprocessed and normalized image tensor, and a direct spatial overlay comparing the manual expert annotation (ground truth, highlighted in green/solid boundaries) against the 2D U‐Net predicted mask (highlighted in red/dashed boundaries). Row 1 (Cohort 1) displays the multiclass delineation of healthy anatomical regions (striatum, neocortex, and hippocampus); Row 2 (Cohort 2) illustrates the segmentation of the ischemic stroke lesion and the surrounding brain parenchyma; Row 3 (Cohort 3) depicts the boundary tracking of the glioblastoma tumor mass.

To ensure scientific transparency, representative edge cases and qualitative failure modes, primarily occurring at the extreme anatomical poles of the brain or in slices with severe artifacts, are documented in Supplementary Figure S1. Finally, to showcase the spatial consistency of the 2D slice‐by‐slice predictions, a comprehensive 3D surface rendering of the structural scans alongside their integrated segmentation masks was generated (Figure 3), underscoring the robust geometric continuity of the proposed framework across diverse pathological phenotypes.

**FIGURE 3:**
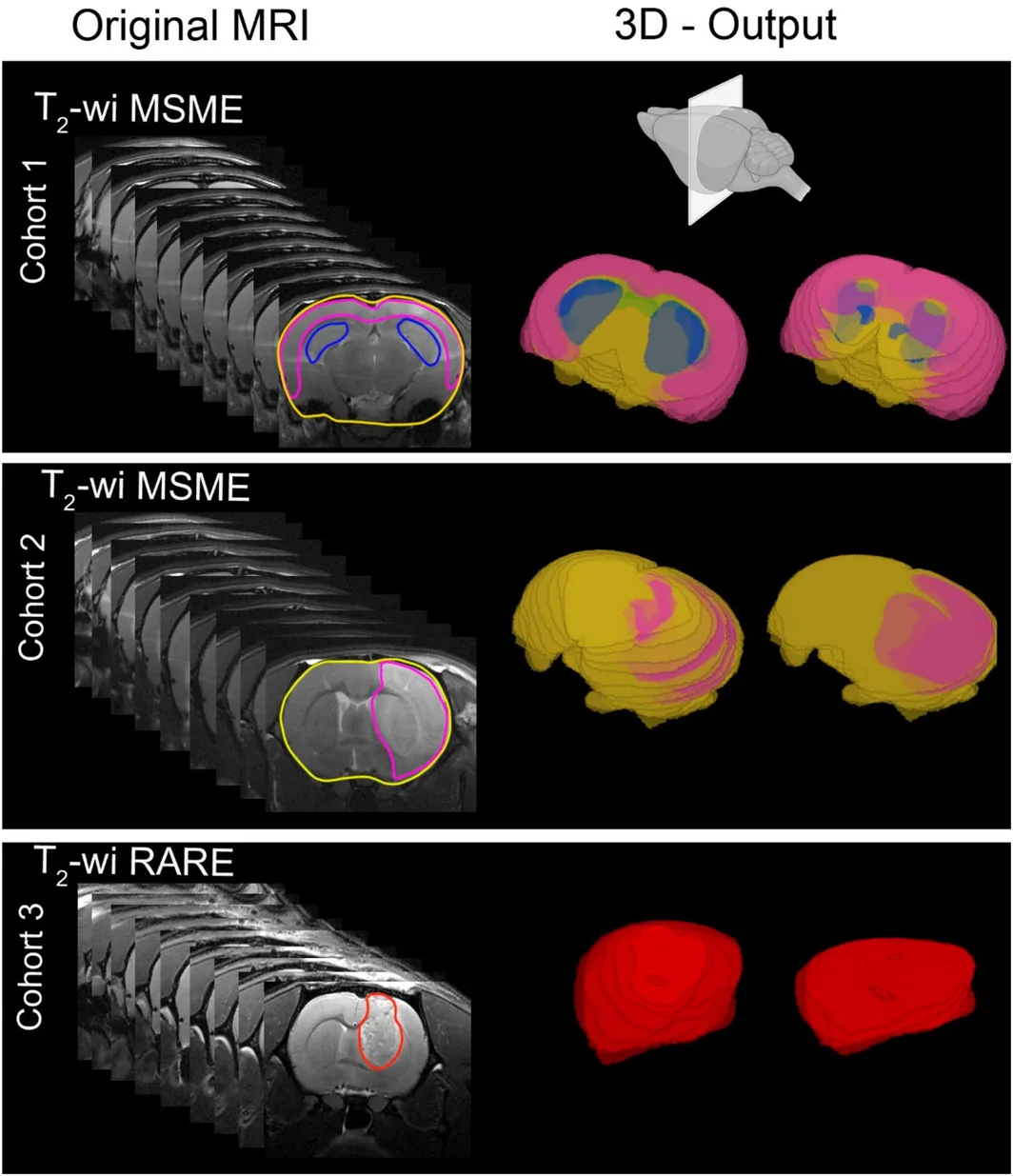
Volumetric 3D surface rendering of the structural MRI scans and automated segmentations. Spatial continuity and geometric accuracy are visualized via 3D multiplanar reconstructions for each experimental target. The semitransparent surface models of the original T_2_‐wi and T_2_‐relaxation scans are overlaid with the integrated 2D U‐Net predicted masks, demonstrating the robust slice‐to‐slice volumetric consistency of the framework across healthy brain structures, ischemic infarct volumes, and infiltrative glioblastoma boundaries.

### 3.5 Quantitative Volumetric Correlation and Methodological Benchmarking

A significant monotonic relationship was observed between the manually annotated ground truth volumes and the automated 2D U‐Net predictions across all experimental conditions (Figure 4a–c). For Cohort 1, the Spearman rank correlation coefficients achieved near‐perfect alignment, yielding ρ=0.99 for the striatum, ρ=0.98 for the neocortex, ρ=0.99 for the hippocampus, and ρ=0.97 for the whole brain. For Cohort 2 (ischemic stroke), robust correlations were obtained for both macro‐structural and physiological parameters, with ρ=0.91 for whole‐brain volume, ρ=0.98 for ischemic lesion volume and ρ=0.82 for localized quantitative T_2_‐relaxation times. Finally, Cohort 3 (glioblastoma) maintained an excellent correlation of ρ=0.92. Across all cohorts and structural classes, these correlations proved to be extremely robust and statistically definitive ($p<10^{-60}$), confirming that the automated framework accurately and reliably replicates expert manual volumetric estimations.

**FIGURE 4:**
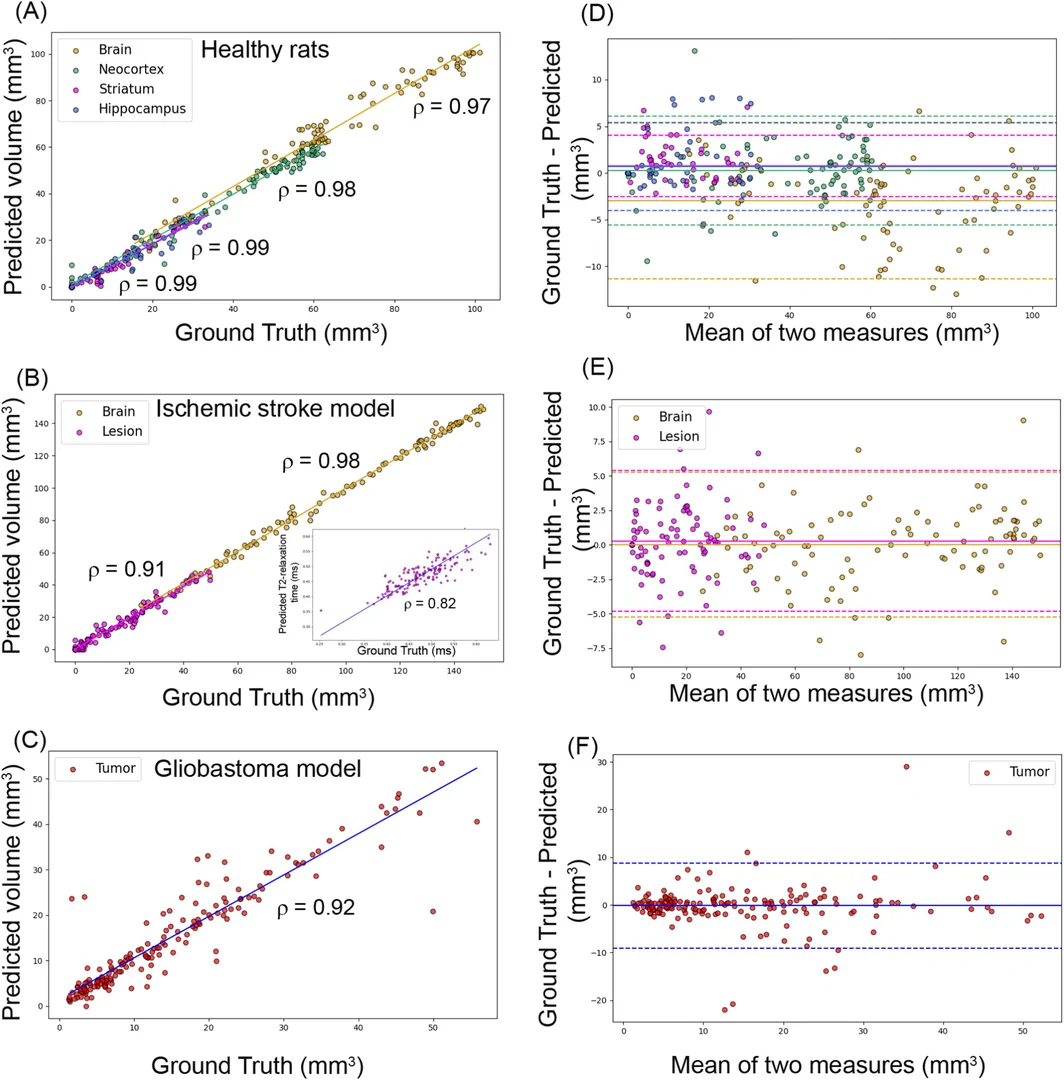
Statistical validation and volumetric agreement between expert ground truth and automated segmentations. (a–c) Spearman rank correlation plots displaying the strong monotonic relationship between the manually annotated volumes and the 2D U‐Net predictions across the three cohorts. (d–f) Bland–Altman plots assessing the degree of agreement and systemic bias between the two methods. In the Bland–Altman analysis, the solid horizontal line represents the mean differences (systemic bias), while the upper and lower dashed lines define the 95% limits of agreement (mean±1.96SD). The inset in (b) displays the quantitative $T_{2}$‐relaxation times of the ischemic lesions, providing additional physiological characterization in relation to the segmented volumes.

To assess systemic bias and limits of agreement, Bland–Altman analyses were executed (Figure 4d–f). The distribution plots revealed no major systematic errors or heteroscedasticity across the cohorts. However, a slight, localized trend toward underestimation was identified within the boundaries of the neocortex and the core ischemic stroke territory. Despite this minor edge‐case compression, the tight distribution within the 95% limits of agreement (mean±1.96·SD) validates the framework's overall clinical and preclinical reliability.

To contextualize our findings within the current state of the art, a comprehensive comparative benchmarking against prominent automated rodent segmentation literature was compiled (Table 4), cross‐referencing modalities, datasets, and performance metrics (DC, HD, SEN, and VR). Compared to established methods, our framework achieved values that were broadly comparable to, and in select structures favorable relative to, those reported in the literature; because these external studies differ from ours in species, imaging protocol, and annotation criteria, the following comparisons are presented for context rather than as evidence of direct superiority: (i) Cohort 1 (healthy brain structures): Our architecture achieved DC of 0.92 ± 0.05 (whole brain), 0.92 ± 0.06 (neocortex), 0.80 ± 0.18 (striatum), and 0.82 ± 0.11 (hippocampus), within the typical literature range reported for comparable structures (DC: 0.91–0.94). (ii) Cohort 2 (ischemic stroke): Unlike most published models that restrict reports to either the isolated lesion or a combined whole‐brain‐lesion mask, our pipeline evaluates the lesion and the healthy brain parenchyma independently. Our metrics were comparable to reported values for the isolated lesion (DC: 0.82 ± 0.23 vs. 0.98; SEN: 0.83 ± 0.24 vs. 0.93) and for the whole‐brain baseline (DC: 0.96 ± 0.02 vs. 0.98; HD: 1.93 ± 1.21 mm vs. 1.86 mm). (iii) Cohort 3 (glioblastoma): the proposed model achieved results comparable to the only available baseline study in rodent 3D/2D tumor segmentation (DC: 0.83 ± 0.16 vs. 0.87; HD: 0.96 ± 0.91 mm vs. 5.9 mm; VR: 1.1 ± 0.59 vs. 0.82), rather than a categorical superiority, given the differences in species, tumor model, imaging protocol, and annotation methodology between studies. These findings support the feasibility and adequacy of the proposed framework for fully automated, high‐throughput preclinical neuroimaging pipelines, within the scope and limitations discussed above.

**TABLE 4:** Comparison of performance across different datasets and a review of the literature.

Cohort 1: Healthy rats									
Dataset	Methods	Dim	Regions	DC	SEN	HD (mm)	VR	Training/Inference time (h/s)	Ref.
Rats	U‐Net	2D	Striatum	0.80	0.75	2.16	0.89	3.9/0.30	This study
			Neocortex	0.92	0.92	0.83	0.99		
			Hippocampus	0.82	0.81	1.50	0.91		
			Brain	0.92	0.94	1.19	1.05		
Mice	U‐Net	2D	Average	0.92	—	—	—	12/0.35	[23]
Mice	MU‐Net	2D	Striatum	0.91	—	—	—	−/−	[24]
			Cortex	0.94	—	—	—		
			Brain mask	0.97	—	—	—		

Cohort 2: Stroke model									
Dataset	Methods	Dim	Regions	DC	SEN	HD (mm)	VR	Training/inference time (h/s)	Ref.
Rats	U‐Net	2D	Lesion	0.82	0.83	1.03	0.99	3.5/0.19	This study
			Brain	0.96	0.96	1.93	1.00		
Rats	RU‐Net	2D	Skull stripping	0.98	0.98	—	—	−/−	[25]
Rats	MedicDeepLabv3+	3D	Hemisphere	0.95	0.97	1.86	—	16/0.40	[26]
Mice	U‐Net	3D	Lesion	0.92	0.93	—	—	100/0.45	[27]
Rats	RatLesNetv2	3D	Lesion	0.81	—	3.33	—	−/−	[28]
Mice	Active contours	3D	Lesion	0.79–0.98	—	—	—	−/4	[29]

Cohort 3: Glioblastoma model									
Dataset	Methods	Dim	Regions	DC	SEN	HD (mm)	*V*_R_	Training/inference time (h/s)	Ref.
Rats	U‐Net	2D	Tumor	0.83	0.85	0.96	1.1	48/0.19	This study
Rats/Mice	U‐Net	3D	Tumor	0.85–0.87	—	5.9	0.82	−/−	[30]

Abbreviations: DC, dice coefficient; Dim, dimension; HD, Hausdorff distance; SEN, sensitivity; VR, volume ratio.

## 4 Discussion

The findings of this study demonstrate the efficacy and versatility of the proposed 2D U‐Net framework for the automated semantic segmentation of diverse anatomical and pathological structures in the rodent brain. By validating the architecture across three distinct cohorts using T_2_‐wi and quantitative T_2_‐relaxation maps, we show that a single deep learning pipeline can reliably accommodate the structural complexities of healthy tissues, focal ischemic lesions, and infiltrative neoplastic masses. The architectural and operational paradigm of our approach offers three practical contributions relative to existing preclinical neuroimaging tools, rather than claiming fundamental architectural novelty. First, the framework transitions from restrictive single‐target delineations to a multiclass topology, enabling the simultaneous extraction of multiple internal brain regions in healthy cohorts, as well as the concurrent, independent segmentation of ischemic cores and total brain parenchyma. Second, the pipeline achieved spatial accuracy metrics that were comparable to, and in some structures favorable relative to, values reported for state‐of‐the‐art methods when evaluated on independent testing datasets, although differences in species, imaging protocol, and annotation criteria across studies preclude a direct claim of superiority. Third, by optimizing a 2D‐based fully convolutional network rather than computationally demanding 3D configurations, we achieved a drastic reduction in both training overhead and active inference time (0.2–0.3 s per slice) without sacrificing volumetric consistency.

Furthermore, our approach achieves higher accuracy in small and anatomically complex regions, such as the hippocampus and striatum, maintains consistent performance across subjects with ischemic stroke or glioblastoma, and produces segmentations that are more reproducible in a preclinical setting. While methods such as segment‐anything‐models (SAM) are versatile and data‐efficient, they can sometimes undersegment or oversegment these regions, particularly in pathological brains. These considerations highlight the robustness and reliability of our approach in comparison to existing methods.

To evaluate the generalizability of the architecture, the 2D U‐Net was benchmarked against a large, highly heterogeneous multistudy dataset compiling 5847 images (partitioned into 4678 slices for training and 1169 slices for independent testing). Because these image tensors were harvested across historical studies encompassing diverse longitudinal postsurgical time points and shifting operational MRI acquisition parameters, the evaluation matrix presents realistic intrastudy multiprotocol variability. The network's sustained accuracy across this heterogeneous, animal‐level‐split repository is consistent with reasonable structural adaptability within the range of protocol variability captured by our own cohorts. However, no externally acquired dataset from a different scanner, laboratory, or acquisition protocol was evaluated, so a claim of resilience to protocol‐induced domain shift beyond the range represented in our training and test data would not be supported by the current evidence; this remains an important direction for future external validation.

Four key metrics comprehensively assess the segmentation performance of our proposed frameworks. Each metric assesses different aspects of segmentation, such as overall accuracy (DC), boundary precision (HD), model sensitivity (SEN), and volume consistency (VR). These widely accepted metrics enable direct comparisons with other methods and ensure our results align with research standards. Metrics like HD help detect boundary discrepancies, crucial for irregular structures, while Volume ratio ensures consistency and avoids oversegmentation or undersegmentation. By utilizing multiple metrics, the robustness and transparency of the evaluation were enhanced, ensuring reliable and reproducible results across different datasets and cohorts.

While volumetric 3D neural architectures have been widely deployed in rodent neuroimaging studies (Table 4), the proposed framework strategically utilizes an optimized 2D U‐Net approach. Although 2D convolutions inherently sacrifice a degree of interslice spatial context along the orthogonal axis, opting for a 2D‐centric pipeline yields profound practical, computational, and translational advantages that outweigh these constraints in routine preclinical research.

From an experimental and translational standpoint, 3D MRI pulse sequences require protracted scan durations, which severely compound the risk of motion artifacts and exponentially extend anesthesia exposure. Minimizing time under volatile anesthesia (e.g., sevoflurane) is a critical refinement in animal welfare, mitigating compounding physiological confounding factors such as core hypothermia, cardiovascular depression, and potential mortality. By demonstrating that a 2D architecture can extract high‐fidelity masks from fast, multislice 2D acquisitions, this framework drastically compresses magnet‐time requirements and simplifies integration into high‐throughput screening protocols.

The 2D U‐Net bypasses the rigorous memory overhead and dense tensor‐reshaping preprocessing workflows required by 3D counterparts. It operates with a lighter architectural footprint, achieving near‐instantaneous inference velocities (0.2–0.3 s per slice) and stable convergence profiles on standard laboratory workstations without requiring high‐end enterprise GPUs. Crucially, as evidenced by our volumetric 3D surface reconstructions (Figure 3), the slice‐by‐slice predictions maintain excellent geometric continuity when stacked, verifying that our 2D approach delivers the operational agility and robust spatial fidelity demanded by modern preclinical environments.

Although not all studies report the training and inference times of their models, our approach generally demonstrates shorter processing durations. In our experiments, the maximum training time required was 48 h, while the shortest, observed in stroke cases, was 3.5 h. The average inference time per image is approximately 0.3 s, which is a typical processing time reported for similar studies [23, 26].

By harvesting image tensors across historical in‐house studies, the training matrix incorporated realistic intraprotocol and interprotocol variability. Exposing the 2D U‐Net to these shifiting image acquisition parameters, structural anatomical micro‐variations, and evolving pathological phenotypes was associated with no clear signs of overfitting within the range of intrastudy variability evaluated, using an animal‐level train/test split. Specifically, the longitudinal pipeline encompassed highly dynamic biological windows, including acute‐to‐chronic phases spanning 1–14 days after stroke induction and progressive oncological stages from 7 to 21 days after tumor implantation. Training the network on this continuous spectrum of tissue alterations, ranging from early cytotoxic edema and localized mass effects to extensive vasogenic edema and infiltrative necrosis, is consistent with the structural adaptability observed within this dataset, although generalization beyond the protocols and pathology stages represented here has not been separately tested.

In contrast to prominent literature on healthy rodent brain segmentation, which primarily restricts its scope to baseline skull stripping or whole‐brain extraction [31, 32, 33, 34], the proposed convolutional neural network framework enables automated, high‐fidelity multiclass segmentation and precise localized volumetric quantification of key internal structures, including the striatum, neocortex, and hippocampus. Furthermore, when benchmarked against current state‐of‐the‐art architectures, our specialized pipeline achieved lesion‐segmentation performance in a broadly comparable range to existing models (spatial overlap, pixel‐wise recall, and boundary localization accuracy), while directly evaluating the whole‐brain parenchyma in the same animals rather than relying on the lesion mask alone. Concurrently, the framework delivered highly competitive and comparable results for whole‐brain parenchymal delineation in terms of DC and SEN scores.

It is well recognized that the major challenges in stroke segmentation in preclinical MRI are: (i) irregular lesion volume and shape, (ii) low resolution compared to clinical imaging data, (iii) fluctuating contrast, and (iv) considerable brain deformation and asymmetry caused by the induced stroke [26, 27, 28, 29]. This lies in 2D/3D automated segmentation methods currently focus primarily on (i) the entire brain, including the lesion based on T_2_‐wi and DWI, (ii) the individual cerebral hemispheres using T_2_‐wi, or (iii) the lesion itself using T_2_‐wi. In the proposed study, both the lesion volume and the whole brain volume were simultaneously quantified through 2D multiclass segmentation. Conducting a longitudinal study by imaging the same animals at multiple time points would allow for dynamic evaluation of disease progression, such as tumor growth or stroke recovery. Furthermore, the integration of T_2_‐relaxation maps enables the extraction of T_2_ relaxation times within the ischemic region. This additional quantitative parameter offers deeper insight into tissue changes over time, contributing to a more comprehensive understanding of ischemic stroke evolution.

To date, literature exploring automated rodent brain tumor segmentation remains profoundly scarce, with only a single pivotal study introducing 3D U‐Net models for this task [30]. That volumetric framework relied heavily on multimodal, contrast‐enhanced 3D MRI inputs, including baseline T_1_‐wi, contrast‐enhanced T_1_‐weighted (CE‐T_1_‐wi), and T_2_‐wi, to delineate tumor‐bearing brains and neoplastic lesions. Remarkably, despite operating within a lower dimensionality and relying exclusively on standard 2D T_2_‐wi structural inputs, our optimized 2D architecture achieved DC, HD, and VR values in a broadly comparable range to this multimodal 3D baseline (DC: 0.833 ± 0.164 vs. 0.87; HD: 0.960 ± 0.913 mm vs. 5.9 mm; VR: 1.099 ± 0.588 vs. 0.82), despite relying on a single, lower‐dimensional imaging sequence rather than multiple contrast‐enhanced 3D acquisitions. This suggests that, with adequate preprocessing and architectural optimization, a lighter 2D framework can extract clinically relevant infiltrative tumor features without the data‐gathering and computational burdens inherent to multisequence 3D networks; however, given the differences in species, tumor model, and imaging protocol between the two studies, these results should be interpreted as comparable performance rather than as evidence of superiority. Nevertheless, the scientific necessity of expanding algorithmic diversity and compiling highly heterogeneous multicenter datasets is fully recognized. By openly releasing our comprehensive, multicohort repository (https://github.com/NOBEL‐MRI/Rat‐Datasets), we actively lower the entry barriers for benchmarking in preclinical neuro‐oncology, providing a foundational resource to foster global standardization and collaborative advancements in automated rodent brain segmentation.

In recent years, quantitative image analysis has emerged as a cornerstone of preclinical translational research. Within this paradigm, radiomics enables the high‐throughput, automated extraction of high‐dimensional imaging features, including advanced shape descriptors, first‐order intensity distributions, and complex textual heterogeneity patterns, offering a noninvasive window into underlying pathophysiology and microstructural tissue niche [4, 5, 6, 7]. However, the cryptographic mathematical nature of radiomic features renders them hypersensitive to initial boundary definitions; even minor structural variations in ROI delineation caused by interobserver variability can fundamentally destabilize feature extraction and compromise downstream predictive outcomes. By providing an automated, deterministic segmentation alternative, the specialized 2D U‐Net models presented in this study systematically mitigate these human‐induced inconsistencies, safeguarding the spatial robustness and mathematical reproducibility of subsequent radiomic pipelines. Consequently, this architecture serves as a foundational upstream engine. When integrated with future advancements in radiomic profiling and machine learning, this pipeline can be seamlessly scaled to support automated molecular classification, noninvasive grading, and longitudinal prognostic modeling in rodent frameworks. Ultimately, bridging the gap between automated spatial delineation and robust feature extraction accelerates the validation and translation of noninvasive imaging biomarkers from preclinical discovery to clinical workflows.

Beyond the aggregate performance metrics reported above, a closer examination of the failure cases documented in Supplementary Figure S1 points to several recurring failure mechanisms. First, segmentation errors were more frequent in small, low‐contrast structures such as the hippocampus and striatum, where boundary ambiguity between adjacent anatomical regions increases the likelihood of misclassification. Second, the infiltrative, poorly circumscribed margins characteristic of glioblastoma were associated with greater boundary uncertainty than the comparatively well‐circumscribed lesions observed in ischemic stroke. Third, segmentation performance may differ between early (24 h after stroke, 7 days after tumor implantation) and later (14‐ and 21‐day) disease stages, as evolving edema and necrosis alter tissue contrast over time; a systematic stage‐stratified analysis of this effect was not performed and is left for future work. Fourth, failure cases concentrated at the extreme anatomical poles of the brain and in slices affected by acquisition artifacts, as noted above. Finally, because the network operates on a slice‐by‐slice 2D basis without explicit interslice contextual information, errors can arise at abrupt anatomical transitions between adjacent slices; this limitation is only partially mitigated by the spatial continuity observed in the 3D surface reconstructions (Figure 3).

Despite the high performance and algorithmic efficiency demonstrated by the proposed framework, several inherent limitations must be addressed. First, a physical, histology‐based ground truth was not explicitly acquired for this study; instead, expert manual segmentations derived from multislice MRI served as the reference standard. However, it is critical to emphasize that the underlying manual annotation protocols utilized by our expert raters have been rigorously validated against co‐registered histological slices in prior foundational literature, where a near‐perfect spatial correlation was established [35]. All segmentations were performed by a single observer per cohort (SOE or SFR), without independent double annotation of the same case; consequently, no interrater agreement figure can currently be calculated retrospectively without additional manual re‐annotation. We acknowledge this explicitly as a study limitation, and we qualify the claim regarding “reduced interobserver variability” so that it is presented not as a quantified advantage but as a design rationale: a deterministic, automated method removes, by construction, interannotator variability in routine use, even though this has not been measured here against a second independent annotator. As partial mitigation, we rely on the previously cited validation of our manual annotation protocol against co‐registered histology [35]. Second, a direct, image‐for‐image architectural comparison against alternative external deep learning models was structurally restricted due to the extreme scarcity of open‐source rodent repositories, the lack of standardized 2D preclinical models, and restricted software access. We mitigated this benchmark barrier by deploying a comprehensive multiparametric statistical suite—including HD and Bland–Altman agreement analyses, to rigorously evaluate our outputs. Consequently, our newly released repository serves as an easily accessible, clearly parameterized, and 2D‐compatible baseline designed specifically to lower entry barriers for future benchmarking initiatives. Third, the networks were optimized exclusively using $T_{2}$‐weighted imaging and quantitative $T_{2}$‐relaxation maps acquired in rats. Therefore, the current weights are strictly eligible for datasets matching these exact imaging dimensions. While $T_{2}$ sequences represent the standard‐of‐care baseline in preclinical neuro‐oncology and ischemia, ongoing architectural scaling aims to incorporate multiparametric sequences (such as Diffusion‐Weighted Imaging) and extend the network's generalizability to mouse frameworks. Fourth, while formal mathematical evaluation under simulated adversarial stress conditions (e.g., additive Gaussian noise, synthetic motion artifacts, or artificial bias fields) was not explicitly executed, the model's out‐of‐sample resilience was inherently tested by the realistic multiprotocol variability of our multistudy dataset. The architecture successfully maintained stable, high‐fidelity segmentation metrics across scans displaying fluctuating signal‐to‐noise ratios, varying intensities, and interoperator acquisition differences. Finally, although the current pipeline is executed via a command‐line Python infrastructure, ongoing software engineering efforts focus on developing a graphical user interface (GUI). This standalone application is designed to streamline input ingestion, automate inference execution, and allow for rapid manual touch‐ups, facilitating the direct integration of these trained models into routine, nontechnical preclinical laboratory workflows.

In conclusion, this study demonstrates the efficacy of tailored 2D U‐Net architectures for the automated segmentation and volumetric characterization of healthy brain structures, ischemic stroke territories, and glioblastoma tumors in rat models utilizing T_2_‐weighted MRI.

By shifting from traditional single‐target masks to a robust multiclass paradigm, the developed framework enables the reliable, simultaneous extraction of critical quantitative metrics, including: (i) absolute volumes and parenchymal percentages of healthy internal brain regions (striatum, neocortex, and hippocampus); (ii) ischemic core volumes and percentages coupled with localized quantitative T_2_‐relaxation times; and (iii) absolute glioblastoma tumor boundaries.

Compared to existing state‐of‐the‐art literature, our framework achieves competitive geometric accuracy and robustness across diverse pathological phenotypes (noting that generalizability was evaluated within the acquisition parameters of our multicohort dataset, while external protocols remain to be tested). By replacing manual tracing with an automated, deterministic approach, the pipeline eliminates interrater and intrarater variability. Combined with near‐instantaneous execution windows, it provides a reliable, scalable, and reproducible resource to support high‐throughput translational neuroimaging research.

To foster open‐science compliance and facilitate standardized preclinical benchmarking, the complete parameterized dataset and computational pipeline are publicly accessible at https://github.com/NOBEL‐MRI/Rat‐Datasets.

## Author Contributions

Organization and design of the study (R.I.R., P.H., and S.O.E.). Data acquisition and processing (S.O.E., M.L.A.A., P.G.A., A.S.V., M.B.B., M.T.A.B., and S.F.R.). Statistical analysis and graphical presentation (J.C. and R.I.). Manuscript drafting (J.C., R.I.R., P.H., M.L.A.A., and C.M.O.). Critical revision and execution of the project (S.O.E., A.J.M., J.P.A., and J.M.F.). Supervision, review, and critique (J.C., C.A.O., M.L.A.A., A.J.M., J.P.A., J.M.F., S.F.R., and C.M.O.). All authors read, reviewed, and agreed the manuscript version.

## Funding

This research was funded by the Spanish Ministry of Science and Innovation (PDC2021‐121455‐I00 and PID2022‐141258OB‐I00), Xunta de Galicia (Axencia Galega de Innovación) (IN607A2022‐03 and IN607A2026‐06), Instituto de Salud Carlos III (ISCIII) (ISCIII/PI21/01256/, PI24/00813, DTS23/00103 and AC21_2/00014), co‐financed by the European Union. Furthermore, R. Iglesias‐Rey (CP22/00061) from the Miguel Servet Program of Instituto de Salud Carlos III and Co‐financed by the EU. M. Bazarra‐Barreiros (FI22/00200) PFIS Researcher of Instituto de Salud Carlos III. C Amodeo‐Oblitas from the Rio Hortega and Juan Rodes Program of Instituto de Salud Carlos III (CM23/00173 and JR25/00032). P. García‐Acevedo thanks to Axencia Galega de Innovación (Spain) for his Posdoctoral Grant (“Axudas de apoio á etapa de formación posdoutoral” ‐ IN606B‐2024.1). This work, integrated into the framework of PERTE for Cutting‐edge Health, has been co‐financed by the Spanish Ministry of Science, Innovation and Universities with funds from the European Union NextGenerationEU, from the Recovery, Transformation and Resilience Plan (PRTR‐C17.I1) and from the Autonomous Community of Galicia within the framework of the Biotechnology Plan Applied to Health. The sponsors did not participate in the study design, collection, analysis, or interpretation of the data, in writing the report, or in the decision to submit the paper for publication.

## Ethics Statement

All experimental animal procedures were conducted under procedure numbers: 15011/2021/002, 15011/2021/003, 15011/2023/002, and 15011/2021/001 approved by the Animal Care Committee, according to European Union Rules and the Spanish regulation (2010/63/EU and RD53/2013). The study was reported in accordance with ARRIVE guidelines (https://arriveguidelines.org).

## Supporting information


**Table S1:** Dice values by tercile are shown for all four structures (*n* = 6 test animals in total, 2 per tercile). No consistent, monotonic size‐performance relationship was observed for any structure, attributed to the narrow range of structure‐volume variation across animals in this cohort, unlike pathological lesions. With only *n* = 2 per tercile, these results are exploratory. *n* = 7 test animals in total; terciles of two to three animals each. Performance is consistently lower and more variable in the small‐lesion tercile than in medium or large lesions. *n* = 33 test animals; 11 animals per tercile. As with Cohort 2, Dice is lower and more variable in the small‐lesion tercile than in medium or large lesions; Hausdorff distance shows a lower mean but a wider spread for small lesions, reflecting a mix of near‐perfect and missed detections at this size. Volume ratio variability is likewise concentrated almost entirely in small lesions.
**Figure S1:** Example qualitative examples of segmentation failure cases. Shown are the original MRI, the normalized MRI, and a comparison between the semiautomated segmentation (used as the ground truth) and the predicted mask. In the first two cases, the model tends to overestimate or underestimate small structures such as the hippocampus. For glioma and ischemic stroke, examples demonstrate that due to regional ambiguity and the inherent difficulty of manual annotation, the model does not achieve perfect segmentation (red arrows indicate the regions of interest). These cases highlight both the challenges of segmenting complex or small brain structures and the limitations of current automated methods in ambiguous pathological regions.

## Data Availability

The datasets generated and/or analyzed during the current study are available in the Github repository (https://github.com/NOBEL‐MRI/Rat‐Datasets). Code is available from authors based on reasonable request and with signed data shared agreement between institutions.
