# Supplementary material for: 2D U‐Net Framework for Multiclass Automated Segmentation of Healthy Structures, Ischemic Stroke, and Glioblastoma in the Rat Brain
Source: NMR Biomed. 2026 Nov 4;39(12):e70432. doi: 10.1002/nbm.70432 (PMC13736198; doi:10.1002/nbm.70432)
Supplement: Supplementary file 1 [file NBM-39-e70432-s001.docx]

**2D U-Net Framework for Multi-Class Automated Segmentation of Healthy Structures, Ischemic Stroke, and Glioblastoma in the Rat Brain**

**Supplementary Table 1:** Dice values by tercile are shown for all four structures (n = 6 test animals in total, 2 per tercile). No consistent, monotonic size-performance relationship was observed for any structure, attributed to the narrow range of structure-volume variation across animals in this cohort, unlike pathological lesions. With only n = 2 per tercile, these results are exploratory. n = 7 test animals in total; terciles of 2–3 animals each. Performance is consistently lower and more variable in the small-lesion tercile than in medium or large lesions. n = 33 test animals; 11 animals per tercile. As with Cohort 2, Dice is lower and more variable in the small-lesion tercile than in medium or large lesions; Hausdorff Distance shows a lower mean but a wider spread for small lesions, reflecting a mix of near-perfect and missed detections at this size. Volume Ratio variability is likewise concentrated almost entirely in small lesions.

| **Cohort 1: Healthy rats (stratified by structure-volume tercile)** | | | | |
| --- | --- | --- | --- | --- |
| **Structure** | **Small tercile Dice** | **Medium tercile Dice** | **Large tercile Dice** | **Volume range (mm³)** |
| Striatum | 0.803 ± 0.169 | 0.821 ± 0.151 | 0.773 ± 0.218 | 83 – 106 |
| Hippocampus | 0.831 ± 0.118 | 0.816 ± 0.106 | 0.827 ± 0.094 | 108 – 115 |
| Neocortex | 0.925 ± 0.044 | 0.927 ± 0.045 | 0.919 ± 0.075 | 500 – 569 |
| Brain | 0.900 ± 0.062 | 0.923 ± 0.038 | 0.924 ± 0.044 | 840 – 942 |

| **Cohort 2: Ischemic stroke, Lesion (stratified by lesion-volume tercile)** | | | | | |
| --- | --- | --- | --- | --- | --- |
| **Lesion-size tercile** | **n** | **Dice** | **Sensitivity** | **HD (mm)** | **VR** |
| Small | 3 | 0.783 ± 0.240 | 0.816 ± 0.263 | 1.091 ± 0.673 | 1.019 ± 0.033 |
| Medium | 2 | 0.827 ± 0.193 | 0.828 ± 0.208 | 0.971 ± 0.895 | 0.963 ± 0.041 |
| Large | 2 | 0.863 ± 0.202 | 0.855 ± 0.218 | 1.000 ± 1.047 | 0.966 ± 0.039 |

| **Cohort 3: Glioblastoma, Tumor (stratified by lesion-volume tercile)** | | | | |
| --- | --- | --- | --- | --- |
| **Lesion-size tercile** | **n** | **Dice** | **HD (mm)** | **VR** |
| Small | 11 | 0.765 ± 0.215 | 0.709 ± 0.798 | 1.28 ± 0.98 |
| Medium | 11 | 0.848 ± 0.133 | 0.862 ± 0.967 | 1.04 ± 0.11 |
| Large | 11 | 0.844 ± 0.158 | 1.084 ± 0.891 | 0.98 ± 0.11 |

**Supplemental Figure S1:** Example qualitative examples of segmentation failure cases. Shown are the original MRI, the normalized MRI, and a comparison between the semi-automated segmentation (used as the ground truth) and the predicted mask. In the first two cases, the model tends to over- or under-estimate small structures such as the hippocampus. For glioma and ischemic stroke, examples demonstrate that due to regional ambiguity and the inherent difficulty of manual annotation, the model does not achieve perfect segmentation (red arrows indicate the regions of interest). These cases highlight both the challenges of segmenting complex or small brain structures and the limitations of current automated methods in ambiguous pathological regions.
